# Differential Adenosine Signaling and Effects of Acute Caffeine Exposure on Alternative Stress Coping Styles in Zebrafish (*Danio rerio*)

**DOI:** 10.1002/brb3.71445

**Published:** 2026-04-29

**Authors:** Sydney E. Klucas, Ryan Y. Wong

**Affiliations:** ^1^ Department of Biology University of Nebraska at Omaha Omaha Nebraska USA

**Keywords:** adenosine pathway, anxiety, caffeine, stress coping styles, zebrafish

## Abstract

**Purpose:**

Changes within neurotransmitter systems are associated with variation in anxiety‐related behavior. The adenosine signaling pathway has been linked to anxiety, and caffeine has been utilized as a modulator. However, studies have not considered the impact of an individual's stress coping style (e.g., proactive, reactive) and the corresponding differences in neuromolecular signaling that can influence behavioral responses.

**Methods:**

To assess the role of adenosine signaling, we acutely treated reactive and proactive zebrafish with 50 mg/L caffeine and evaluated anxiety‐like behavior using a novel tank diving test. We then quantified whole‐brain gene expression of genes representing distinct parts of the adenosine signaling pathway: adenosine receptors A1B, A2Aa, A2Ab, and A2B (*adora1b*, *adora2aa*, *adora2ab*, and *adora2b*, respectively) and enzymes adenosine deaminase (*ada*) and ecto‐5′‐nucleotidase (*nt5e*).

**Findings:**

Individuals with the reactive coping style had higher anxiety‐related behavior (lower composite stress behavior score, more time spent frozen, less transitions to the top half, swam less distance, and swam slower) than the proactive coping style, and all females had higher anxiety‐related behavior (lower composite stress behavior score, more time spent frozen, swam less distance, and swam slower) than males. Surprisingly, caffeine treatment decreased anxiety‐related behavior (swam a higher distance and swam faster) compared to controls. Caffeine reduced anxiety behavior (higher composite stress behavior score, swam a higher distance, and swam faster) in reactive males only. We also observed significant differential gene expression within the adenosine signaling pathway between the reactive and proactive strains, where reactive zebrafish expressed higher levels of adenosine receptors A1B, A2Ab, A2B, and *ada*, and lower levels of adenosine receptor A2Aa than proactive zebrafish.

**Conclusion:**

These findings indicate that variation in adenosine signaling between the stress coping styles and sexes may be contributing to differences in anxiety‐related behavior.

## Graphical abstract

Acute caffeine exposure exerts coping style‐ and sex‐dependent effects on anxiety‐like behavior in zebrafish, with reactive individuals, particularly males, showing the greatest sensitivity. The accompanying changes in adenosine receptor and adenosine deaminase expression demonstrate a possible molecular mechanism underlying these behavioral differences, highlighting the adenosine signaling pathway as a promising target for understanding individual variability in anxiety.

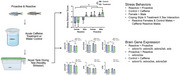

## 1 Introduction

Animals encounter many stressful events, such as obtaining resources among competitors or avoiding predators, and often utilize strategies to successfully overcome these stressors. The resulting physiological and behavioral changes to a stressor are mediated in part by the sympathetic–adrenal–medullary and hypothalamic–pituitary–adrenal (HPA) axes (Vreeburg et al. 2010; Shimada‐Sugimoto et al. 2015). Exaggerated or inappropriate responses to stressors are symptoms of anxiety‐related disorders, and it is hypothesized that dysregulation of neurotransmitter signaling pathways can facilitate the progression of the disorders (Martin et al. 2010). Specifically, variation in the adenosine neurotransmitter system may increase susceptibility to anxiety disorders (Van Calker et al. 2019; Alasmari 2020).

Adenosine is a neuromodulator that has a crucial role in cellular signaling. Within the adenosine pathway of the central nervous system, adenosine is a predominantly inhibitory neuromodulator that binds to the A1, A2A, A2B, and A3 adenosine receptors. As G protein‐coupled receptors (GPCRs), adenosine A1 and A3 receptors are coupled to inhibitory G_i/o_ proteins. A2A receptors are coupled to G_s_ and A2B receptors are coupled to G_s/q_ proteins, causing inhibitory or excitatory effects on several downstream signaling pathways (Antonioli et al. 2019; Sheth et al. 2014). The binding of adenosine to these receptors is essential for the sleep–wake cycle, immune responses, inflammation, and several other downstream effects (Bjorness and Greene 2009; Allard et al. 2020). Adenosine levels are highly regulated through degradation and production by multiple enzymes. Two of the main extracellular enzymes within the pathway include ecto‐5′‐nucleotidase (*nt5e*, also known as CD73), which produces adenosine by hydrolyzing adenosine monophosphate (AMP), and adenosine deaminase (*ada*), which catalyzes the deamination of adenosine into inosine. The adenosine signaling pathway has been implicated in anxiety, wherein both adenosine concentration and adenosine receptor interactions are altered (Zimmermann et al. 2016; Piato, Capiotti, et al. 2011; Childs et al. 2008; Yacoubi, Ledent, Ménard, et al. 2000; Jain et al. 1995; Maximino et al. 2011; Rosa et al. 2018; Hughes and Hancock 2016; Bai et al. 2014). Adenosine levels increase in the brain after chronic stress (Zimmermann et al. 2016). Both ecto‐ and cytosolic *ada* activity decrease following a stressor (Zimmermann et al. 2016; Piato, Capiotti, et al. 2011; Bai et al. 2014), which may contribute to an increase in adenosine levels. Administration of an adenosine derivative in mice decreased both anxious behavior and *ada* activity and increased adenosine levels in the cortex and hypothalamus (Bai et al. 2014; Huang et al. 2014). Caffeine is another compound that can alter adenosine signaling through unbiased competitive inhibition of adenosine receptors A1 and A2A. Genetic variation in adenosine receptors A1 and A2A is associated with panic disorder and anxiety following caffeine consumption in humans (Childs et al. 2008; Hohoff et al. 2010; Fulton et al. 2018; Alsene et al. 2003; Rogers et al. 2010), and antagonism of adenosine receptors by caffeine has been found to be implicated in anxiety behavior (Yacoubi, Ledent, Ménard, et al. 2000; Jain et al. 1995; Maximino et al. 2011; Rosa et al. 2018; Hughes and Hancock 2016; Li et al. 2024). Behavioral and physiological effects of caffeine vary with the treatment dose, where lower doses induce stimulatory effects while higher doses induce anxiety behavior (Yacoubi, Ledent, Ménard, et al. 2000; Egan et al. 2009; Tran et al. 2017; Svenningsson et al. 1997). Although different populations of zebrafish and rats differ in their anxiogenic responses (Rosa et al. 2018; Hughes and Hancock 2016), it is not yet known if genetic differences in the adenosine pathway are responsible in part for altered anxiety‐like behavior.

Zebrafish (*Danio rerio*) are a commonly used animal model for understanding the molecular and pharmacological bases of anxiety due to their ease of maintenance and their highly conserved genetic and physiological features with mammals and other species (Egan et al. 2009; Rico et al. 2011). To facilitate identifying molecular mechanisms of stress‐ and anxiety‐like behaviors, prior studies have utilized zebrafish strains selectively bred to display opposing stress coping styles: proactive and reactive (Wong et al. 2012, 2013, 2015, 2019; Goodman and Wong 2020). Across different stress assays and time, the reactive zebrafish strain displays higher levels of anxiety‐like behaviors and has a faster whole‐body cortisol response compared to the proactive zebrafish (Wong et al. 2012, 2013, 2015, 2019; Goodman and Wong 2020). Further, the two zebrafish strains show distinct baseline neurotranscriptome profiles, including differences in expression within several neurotransmitter pathways, such as dopamine, serotonin, GABA, and adenosine (Wong et al. 2015; Wong and Godwin 2015). Receptors and enzymes within the zebrafish adenosine pathway are homologous to those of mammals (Nabinger et al. 2020), and their presence in the zebrafish brain is well‐documented (Senger et al. 2004; Rosemberg et al. 2007; Rosemberg et al. 2008; Boehmler et al. 2009; Capiotti et al. 2011). Within the adenosine pathway, the proactive strain has significantly higher whole‐brain expression of *ada* and *nt5e* compared to the reactive strain at baseline (Wong et al. 2015). In addition to stress coping style, sex differences have been documented in anxiety‐related behavior and adenosine signaling; females generally exhibit higher anxiety than males across species (Li et al. 2024; Wong et al. 2012, McLean et al. 2011; Biedermann et al. 2017; Mariscal et al. 2023; Bishnoi et al. 2021) and demonstrate sex‐specific variation in the adenosine system (Borgus et al. 2020; Pierling et al. 2021; Yang et al. 2007; Pires et al. 2009). There is limited understanding of how stress coping styles and sex interact to affect anxiety behavior mediated by adenosine signaling.

In the present study, we investigated the effects of acute caffeine treatment on anxiety‐related behavior and gene expression within the adenosine signaling pathway in proactive and reactive strains of zebrafish. We evaluated stress behaviors using the novel tank diving test (NTDT) and quantified whole‐brain gene expression of adenosine receptors A1B, A2Aa, A2Ab, and A2B (*adora1b*, *adora2aa*, *adora2ab*, and *adora2b*, respectively) as well as the *ada* and *nt5e* enzymes. We hypothesized that acute caffeine exposure will increase anxiety‐like behavior in both coping styles with an amplified anxiogenic response in the reactive strain. At baseline, we predicted that the reactive strain would have increased expression of adenosine receptors and *ada* compared to the proactive strain. As supported by well‐studied mechanisms of receptor antagonists (Svenningsson et al. 1999; Ribeiro and Sebastião 2010; Toth and Shenk 1994), it is also expected that adenosine receptor antagonism by caffeine will downregulate receptor expression regardless of zebrafish strain. Understanding how an adenosine receptor antagonist impacts adenosine signaling between the two coping styles and sexes will provide insight into a mechanism that may explain differences in their anxiety‐related behaviors.

## 2 Methods

### 2.1 Subjects

We used zebrafish (*D. rerio*) strains selectively bred to display the proactive or reactive stress coping style. The strains originated from wild‐caught zebrafish from Gaighata, India, and were then selectively bred based on behavioral response to stress (Wong et al. 2012). The two strains have well‐documented differences in stress‐related responses across several behavioral assays, time, morphology, neuropharmacology, neurotranscriptome analyses, learning and memory, and glucocorticoid responses (Wong et al. 2012, 2013, 2019; Goodman and Wong 2020; Wong and Godwin 2015; Baker et al. 2018; Baker and Wong 2019; Corcoran et al. 2025). In this study, all fish were 9–11 months old when testing began and had been selectively bred for 14 generations. Prior to testing, zebrafish were housed in 40 L mixed‐sex tanks on a custom‐built recirculating water system with solid and biological filtration. System water was held at approximately 27°C with a 700 µS conductivity and a 7.2 pH. Zebrafish were on a 14:10 light/dark cycle and fed twice daily with Tetramin Tropical Flakes (Tetra, USA). All procedures and experiments were approved by the Institutional Animal Care and Use Committee of the University of Nebraska at Omaha (17‐070‐09‐FC).

### 2.2 Caffeine Treatment

We treated proactive and reactive zebrafish with caffeine or vehicle control for 15 min and then measured their stress‐ and anxiety‐related behaviors with a novel tank diving test (NTDT) for 30 min. Sample size included 30 proactive zebrafish (*n* = 15 control, with 6 females and 9 males; *n* = 15 treated, with 5 females and 10 males) and 30 reactive zebrafish (*n* = 15 control, with 6 females and 9 males; *n* = 15 treated, with 7 females and 8 males). For acute treatment, zebrafish were randomly placed in system water with 0 or 50 mg/L caffeine (Sigma Aldrich) for 15 min following previously established protocols (Rosa et al. 2018; Li et al. 2024; Egan et al. 2009). We used 50 mg/L caffeine concentration after conducting a pilot dose–response study where we found 50 mg/L significantly changed stress‐related behavior compared to controls in at least one of the strains (see Figure S1 and Table S2).

### 2.3 Behavioral Assay

After 15 min of caffeine or vehicle control treatment, we subjected individual zebrafish to a novelty stressor with an NTDT, following established procedures (Egan et al. 2009; Wong et al. 2012, 2013, 2019; Cachat et al. 2010). In brief, we used a clear 3‐L trapezoidal tank (15.2 height × 28.3 top × 22.5 bottom × 11.4 cm width; Pentair Aquatic Ecosystems) filled with 2 L of system water. We video‐recorded the fish for 30 min, and the behavior was then quantified using video tracking software (Ethovision Noldus XT Version 17, Wageningen, Netherlands), as previously described (Wong et al. 2012, 2015, 2019; Baker et al. 2018). We quantified behavioral indicators of stress for the first 6 min, including duration (s) in the bottom half of the tank, number of transitions into the top half of the tank, total distance swam (cm), average velocity (cm/s), and time spent frozen (s). The subject was considered frozen if it was moving less than 0.55 cm/s. This time was chosen to ensure we were measuring the response to a novelty stressor without the confounds of habituation (Raymond et al. 2012; Wong, Elegante, et al. 2010) and to maintain comparability with similarly used timeframes in many other published studies (Rosa et al. 2018; Egan et al. 2009; Wong et al. 2012, 2013; Goodman and Wong 2020). Increased time frozen, time spent in the bottom half of the tank, and decreased transitions to the top half are indicators of heightened stress and anxiety (Egan et al. 2009; Wong et al. 2012). Distance swam and average velocity were used to assess locomotor activity. Behavioral testing occurred between 6 and 10 h after light onset. Immediately after the behavior test, we extracted the brains and stored them at −80°C until processing. We determined the sex of each fish by the identification of testes or ovaries on dissection.

### 2.4 Gene Expression Analysis

Using quantitative reverse transcription PCR (qRT‐PCR), we quantified the whole‐brain gene expression of adenosine receptors (*adora1, adora2aa, adora2ab, adora2b*) and enzymes *ada* and *nt5e* with *ef1a* used as an endogenous reference for normalization following established protocols (Wong et al. 2013, 2015; Wong and Godwin 2015). Expression of *ef1a* is stable across sex, developmental stage, chemical treatment, and tissue type in zebrafish (McCurley and Callard 2008). Gene expression analyses were conducted on the whole brain because anxiety involves multiple brain regions (Martin et al. 2010), allowing for analysis of the entire neural network's effect on behavior. In brief, we homogenized the brain tissue using zirconium oxide beads (Bullet Blender, Next Advanced) and then extracted and purified RNA (RNeasy Plus Mini, Qiagen). For each sample, we quantified RNA concentration with a Qubit 3.0 (Life Technologies). For each fish, we used 230 ng of total RNA for cDNA synthesis (SuperScript IV First‐Strand Synthesis System, Invitrogen) and purified the cDNA using Amicon Ultracentrifugal filters (Millipore).

For qRT‐PCR, we utilized the QuantStudio 7 Flex Real‐Time PCR System (Applied Biosystems) using SYBR green detection chemistry (PowerUp SYBR Green Master Mix, Applied Biosystems). We designed all primers on Primer‐Blast (NCBI), except for *ef1a*, which was determined by past studies (Ye et al. 2012; McCurley and Callard 2008). The primer concentration was 5 pmol for every gene except *adora2aa* (1 pmol) and *adora2ab* (2.5 pmol) (Table S1). Reaction parameters for each gene were as follows: 2 min at 50°C, 2 min at 95°C, and 40 cycles of 95°C for 15 s and 60°C for 1 min. We ran each sample in triplicate and quantified expression using the relative standard curve method.

### 2.5 Statistical Analysis

Of the twelve dependent variables, four were not normally distributed even after a log transformation. Therefore, we applied a generalized linear model (GLZM) with the identity link function in SPSS (Version 29) to examine changes in behaviors and normalized gene expressions. Using the GLZM, we investigated the main effects of strain (reactive, proactive), sex (female, male), treatment (50 mg/L caffeine, control), strain×treatment interaction, and strain×treatment×sex interaction on behavior and gene expression. To evaluate the direction of effects, we examined the simple main effects within each GLZM and applied a Benjamini–Hochberg correction when examining the interaction effects (Benjamini and Hochberg 1995). Nonparametric (Spearman's) correlations were also analyzed between each dependent variable, followed by a Benjamini–Hochberg correction (Benjamini and Hochberg 1995). In addition, we used principal component analysis (PCA) with the five behavioral variables to calculate principal component (PC) scores for each individual. This score was also used as a dependent variable in the GLZM as described above.

## 3 Results

### 3.1 Significant Effect of Strain and Sex on Composite Behavioral Scores

**FIGURE 1:**
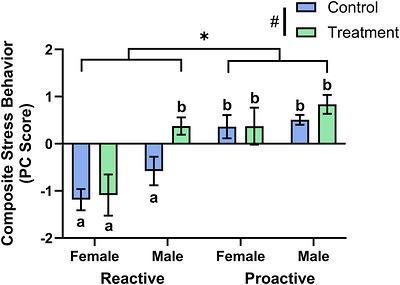
Acute caffeine treatment significantly altered behavior between strains and sexes. For each group, we calculated a stress PC score from distance swam, average velocity, time spent frozen, number of top transitions, and time spent in bottom half. Data shown are mean ± 1 SEM. Significant strain and treatment differences are indicated by an asterisk (*p* ≤ 0.05), while trends are indicated by a # (0.05 < *p* < 0.1). Significant differences between groups are indicated by different lowercase letters (*p* ≤ 0.05).

PCA of the five behavioral variables revealed that all behaviors loaded onto a single component (stress behavior axis), where swim velocity, distance swam, and number of transitions into the top half of the tank loaded positively, while time frozen and time in the bottom half of the tank loaded negatively. The resulting PC scores demonstrate that more negative scores indicate higher stress‐related behaviors. There was a significant main effect of strain on the PC scores (Wald *χ* ^2^ = 40.389, *p* = 2.081 × 10^−10^) with reactive fish having significantly lower scores than proactive fish (Figure 1). There was also a significant main effect of sex on the PC scores (Wald *χ* ^2^ = 13.997, *p* = 0.000183) with female fish having significantly lower PC scores than males (Figure 1). There was a trend of a main effect of treatment on the PC scores (Wald *χ* ^2^ = 3.774, *p* = 0.052), in which caffeine‐treated fish had an increase in PC scores relative to controls. There was also a trend of a strain by treatment by sex interaction effect on the PC scores (Wald *χ* ^2^ = 7.660, *p* = 0.054). Caffeine‐treated reactive females had significantly lower PC scores than caffeine‐treated reactive males (*p* = 2.84 × 10^−5^) and caffeine‐treated proactive females (*p* = 2.21 × 10^−4^). Caffeine‐treated reactive males had significantly higher PC scores than male reactive controls (*p* = 0.00368). Female reactive controls had significantly lower PC scores than female proactive controls (*p* = 7.38 × 10^−5^). Male reactive controls had significantly lower PC scores than male proactive controls (*p* = 6.58 × 10^−4^). There was not a significant strain by treatment interaction effect (Wald *χ* ^2^ = 0.986, *p* = 0.321). For raw behavioral and gene expression data, see Table S3.

### 3.2 Stress Coping Style, Acute Caffeine Exposure, and Sex Alter Discrete Behaviors

**FIGURE 2:**
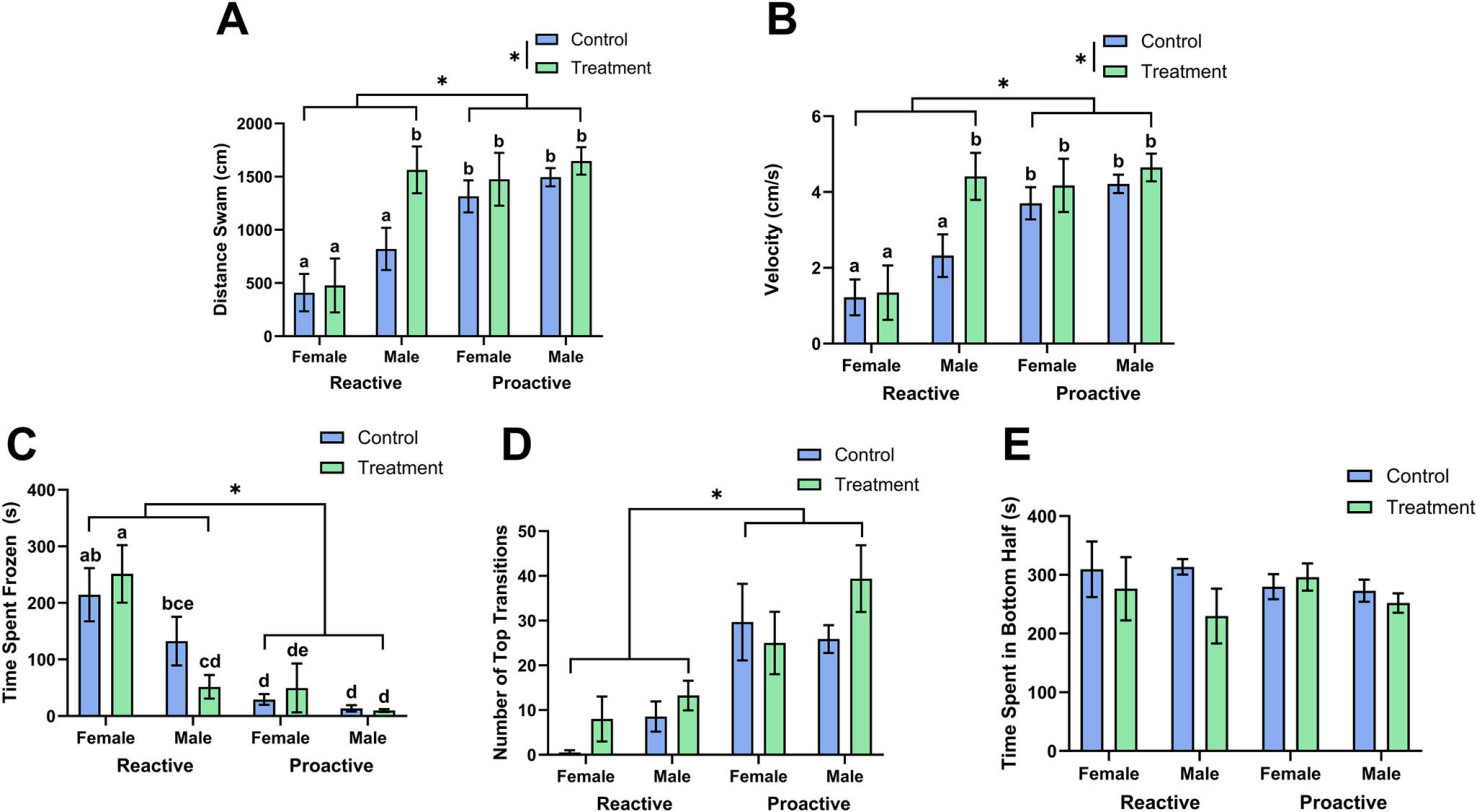
Female zebrafish exhibited higher anxiety‐related behavior than males. We measured distance swam (A), average velocity (B), time spent frozen (C), number of top transitions (D), and time spent in bottom half (E) for each group. Data shown are mean ± 1 SEM. Significant strain and treatment differences are indicated by an asterisk (*p* ≤ 0.05), while significant differences between groups are indicated by different lowercase letters (*p* ≤ 0.05).

There were significant main effects of strain on the number of transitions into the top half (Wald *χ* ^2^ = 37.667, *p* = 8.39 × 10^−10^), time spent frozen (Wald *χ* ^2^ = 42.802, *p* = 6.06 × 10^−11^), swimming velocity (Wald *χ* ^2^ = 28.395, *p* = 9.89 × 10^−8^), and distance swam (Wald *χ* ^2^ = 28.768, *p* = 8.16 × 10^−8^). Compared to proactive fish, reactive fish had less transitions to the top of the tank, spent more time frozen, swam with lower velocity, and swam less distance total (Figure 2). There was not a significant main effect of strain on time spent in the bottom half (Wald *χ* ^2^ = 0.107, *p* = 0.743).

Caffeine treatment significantly increased swimming velocity (Wald *χ* ^2^ = 5.007, *p* = 0.025) and distance swam (Wald *χ* ^2^ = 5.121, *p* = 0.024) compared to control fish. There were not significant main effects of caffeine treatment on time spent in the bottom half (Wald *χ* ^2^ = 1.985, *p* = 0.159), number of transitions into the top half (Wald *χ* ^2^ = 2.074, *p* = 0.150), or time spent frozen (Wald *χ* ^2^ = 0.107, *p =* 0.744). There were no significant strain by treatment interaction effects on behavioral variables (number of transitions into the top half [Wald *χ* ^2^ = 0.053, *p* = 0.819], time spent in the bottom half [Wald *χ* ^2^ = 1.704, *p* = 0.192], time spent frozen [Wald *χ* ^2^ = 0.523, *p* = 0.469], swimming velocity [Wald *χ* ^2^ = 0.884, *p* = 0.347], and distance swam [Wald *χ* ^2^ = 1.010, *p* = 0.315]).

There were significant main effects of sex on time spent frozen (Wald *χ* ^2^ = 16.247, *p* = 5.556 × 10^−5^), swimming velocity (Wald *χ* ^2^ = 13.604, *p* = 2.257 × 10^−4^), and distance swam (Wald *χ* ^2^ = 13.908, *p* = 1.920 × 10^−4^) but not on the time spent in the bottom half (Wald *χ* ^2^ = 1.178, *p* = 0.278) and the number of transitions into the top half (Wald *χ* ^2^ = 2.683, *p* = 0.101). Compared to male fish, female fish spent more time frozen, swam with less velocity, and swam less distance total (Figure 2).

There were significant strain by treatment by sex interaction effects for time spent frozen (Wald *χ* ^2^ = 11.738, *p* = 0.008), swimming velocity (Wald *χ* ^2^ = 9.386, *p* = 0.025), and distance swam (Wald *χ* ^2^ = 9.251, *p* = 0.026). Caffeine‐treated reactive females spent significantly more time frozen, swam less distance, and swam at a lower velocity than caffeine‐treated reactive males (*p* = 1.02 × 10^−6^, *p* = 7.32 × 10^−6^, *p* = 6.93 × 10^−6^, respectively) and caffeine‐treated proactive females (*p* = 1.29 × 10^−5^, *p* = 2.74 × 10^−4^, *p* = 2.47 × 10^−4^, respectively). Female reactive controls spent significantly more time frozen, lower swimming velocity, and swam a lower distance than female proactive controls (*p* = 4.93 × 10^−5^, *p* = 0.00110, *p* = 8.10 × 10^−4^, respectively). Female reactive controls spent significantly more time frozen than male reactive controls (*p* = 0.0491) but did not remain significant after a Benjamini–Hochberg adjustment. Male reactive controls had significantly more time frozen, lower swimming velocity, and swam a lower distance than male proactive controls (*p* = 0.00139, *p* = 0.00229, *p* = 0.00224, respectively). In addition, male reactive controls had significantly lower swimming velocity and swam a lower distance than caffeine‐treated reactive males (*p* = 0.00108, *p* = 0.00109, respectively). Male reactive controls had significantly higher time frozen than caffeine‐treated reactive males (*p* = 0.0350) but did not remain significant after a Benjamini–Hochberg adjustment. There was not a significant strain by treatment by sex interaction effect on time spent in the bottom half (Wald *χ* ^2^ = 1.079, *p* = 0.782) and number of transitions into the top half (Wald *χ* ^2^ = 3.139, *p* = 0.371).

### 3.3 Differential Gene Expression of *adora1b*, *adora2aa*, *adora2ab*, *adora2b*, and *ada* Between Zebrafish Strains

**FIGURE 3:**
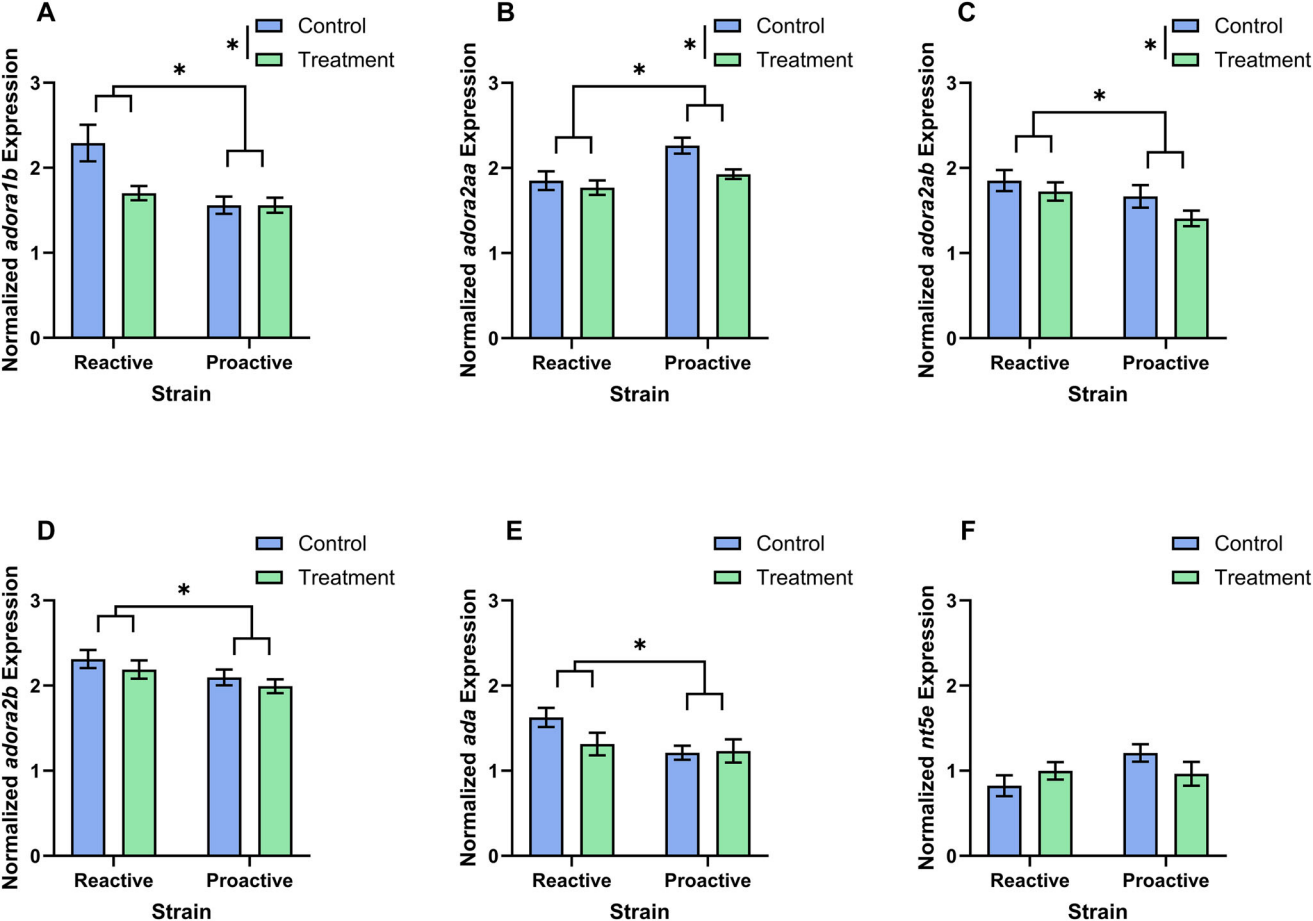
Differential gene expression of *adora1b*, *adora2aa*, *adora2ab*, *adora2b*, and *ada* between zebrafish strains. We measured expression of *adora1b* (A), *adora2aa* (B), *adora2ab* (C), *adora2b* (D), *ada* (E), and *nt5e* (F) for each group. Data shown are mean ± 1 SEM. Significant strain and treatment differences are indicated by an asterisk (*p* ≤ 0.05).

There were significant main effects of strain on expression of *adora1b* (Wald *χ* ^2^ = 13.079, *p* = 0.0003), *adora2aa* (Wald *χ* ^2^ = 11.075, *p* = 0.00088), *adora2ab* (Wald *χ* ^2^ = 6.862, *p* = 0.0088), *adora2b* (Wald *χ* ^2^ = 5.521, *p* = 0.019), and *ada* (Wald *χ* ^2^ = 4.887, *p* = 0.027) but not *nt5e* (Wald *χ* ^2^ = 1.983, *p* = 0.159). Compared to proactive fish, reactive fish had lower expression of *adora2aa* and higher expression of *adora1b*, *adora2ab*, *adora2b*, and *ada* (Figure 3).

There were significant main effects of caffeine treatment on expression of *adora1b* (Wald *χ* ^2^ = 5.015, *p* = 0.025), *adora2aa* (Wald *χ* ^2^ = 4.976, *p* = 0.026), and *adora2ab* (Wald *χ* ^2^ = 4.381, *p* = 0.036) but not adora2b (Wald *χ* ^2^ = 1.078, *p* = 0.299), *ada* (Wald *χ* ^2^ = 1.469, *p* = 0.226), or *nt5e* (Wald *χ* ^2^ = 0.084, *p* = 0.771). Caffeine‐treated fish had lower expression of *adora1b*, *adora2aa*, and *adora2ab* compared to control fish (Figure 3).

There was a significant strain by treatment interaction effect for *adora1b* expression (Wald *χ* ^2^ = 4.952, *p* = 0.026). Reactive control fish had significantly higher expression of *adora1b* than proactive control fish (*p* = 3.27 × 10^−5^) and reactive treated fish (*p* = 0.00136). There was a trend in strain by treatment interaction effects for expression of *ada* (Wald *χ* ^2^ = 3.385, *p* = 0.066) and *nt5e* (Wald *χ* ^2^ = 2.794, *p* = 0.095). Reactive controls had significantly higher expression of *ada* than proactive controls (*p* = 0.00398). Reactive controls had significantly higher expression of *ada* than caffeine‐treated reactive fish (*p* = 0.0286) but did not remain significant after a Benjamini–Hochberg adjustment. Reactive controls had significantly lower expression of *nt5e* than proactive controls (*p* = 0.0285) but did not remain significant after a Benjamini–Hochberg adjustment. There were not significant strain by treatment interaction effects for expression of *adora2aa* (Wald *χ* ^2^ = 0.994, *p* = 0.319), *adora2ab* (Wald *χ* ^2^ = 0.334, *p* = 0.563), and *adora2b* (Wald *χ* ^2^ = 0.057, *p* = 0.811).

There was not a significant main effect of sex on gene expression (*adora1b*: Wald *χ* ^2^ = 2.444, *p* = 0.118; *adora2aa*: Wald *χ* ^2^ = 0.159, *p* = 0.691; *adora2ab*: Wald *χ* ^2^ = 0.001, *p* = 0.977; *adora2b*: Wald *χ* ^2^ = 0.583, *p* = 0.445; *ada*: Wald *χ* ^2^ = 1.324, *p* = 0.250; *nt5e*: Wald *χ* ^2^ = 0.172, *p* = 0.678).

There was a significant strain by treatment by sex interaction effect for *adora2ab* expression (Wald *χ* ^2^ = 10.152, *p* = 0.017). Female reactive control fish had significantly higher expression of *adora2ab* than male reactive control fish (*p* = 0.0104). Female reactive control fish had significantly higher expression of *adora2ab* than female reactive treated fish (*p* = 0.0053) and female proactive controls (*p* = 0.023) but did not remain significant after a Benjamini–Hochberg adjustment. Caffeine‐treated reactive males had a trend of higher expression of *adora2ab* than caffeine‐treated proactive males (*p* = 0.009) but did not remain significant after a Benjamini–Hochberg adjustment.

There was a trend in the strain by treatment by sex interaction effect for expression of *adora2aa* (Wald *χ* ^2^ = 7.176, *p* = 0.067). Male reactive controls had significantly lower expression of *adora2aa* than male proactive controls (*p* = 1.99 × 10^−5^). Male proactive controls had significantly higher expression of *adora2aa* than caffeine‐treated proactive males (*p* = 1.66 × 10^−4^). Female proactive controls had a trend of higher expression of *adora2aa* than male proactive controls (*p* = 0.0448) but did not remain significant after a Benjamini–Hochberg adjustment. There were not significant strain by treatment by sex interaction effects for expression of *adora1b* (Wald *χ* ^2^ = 0.811, *p* = 0.847), *adora2b* (Wald *χ* ^2^ = 4.132, *p* = 0.248), *ada* (Wald *χ* ^2^ = 5.481, *p* = 0.140), and *nt5e* (Wald *χ* ^2^ = 1.007, *p* = 0.799).

### 3.4 Expression of *adora2ab* and *ada* Are Positively Correlated With Anxiety Behavior

**FIGURE 4:**
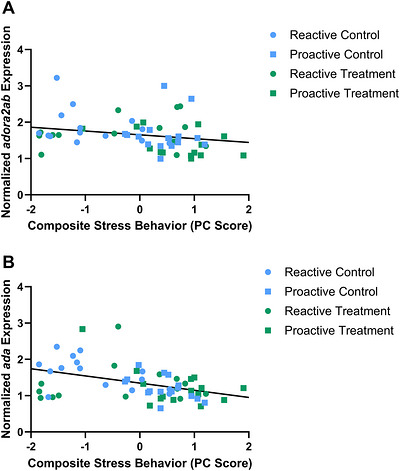
Expression of *adora2ab* and *ada* is correlated with the composite stress behavior scores. Significant correlation between gene expression, *adora2ab* (A) and *ada* (B), and behavioral PC scores within all zebrafish (reactive control, blue circle; proactive control, blue square; reactive treatment, green circle; proactive treatment, green square).

There are significant correlations between the PC score and the expression of *adora2ab* (*p*_Benjamini–Hochberg_ < 0.05, *ρ* = −0.375) and *ada* (*p*_Benjamini–Hochberg_ < 0.05, *ρ* = −0.410), in which the higher the expression, the higher the anxiety behavior (Figure 4). While there was a significant correlation between *adora2b* expression and PC score (*p* = 0.028, *ρ* = −0.285), this relationship did not remain significant after a Benjamini–Hochberg adjustment. There was not a significant correlation between the PC score and expression of *adora1b* (*p* = 0.0916, *ρ* = −0.220), *adora2aa* (*p* = 0.174, *ρ* = 0.178), or *nt5e* (*p* = 0.503, *ρ* = 0.088).

## 4 Discussion

This study investigated how the adenosine signaling pathway and antagonism of the pathway with acute caffeine treatment influence the behavior of proactive and reactive zebrafish strains. We found that reactive zebrafish express higher levels of adenosine receptors A1B, A2Ab, A2B, and *ada* and lower levels of adenosine receptor A2Aa than proactive zebrafish. In addition, acute caffeine exposure differentially affected the stress coping styles and the sexes. The proactive strain behavior was not significantly impacted by caffeine treatment while the reactive strain behavior differed by sex. Following caffeine treatment, female reactive zebrafish exhibited higher anxiety behavior and male reactive zebrafish exhibiting lower anxiety but hyperactive behavior. We also noted that overall female zebrafish had higher anxiety behavior and demonstrated that *adora2ab* and *ada* expression is positively correlated with anxiety behavior. These findings indicate that variation in adenosine signaling may contribute to differences in anxiety‐related behavior between the stress coping styles and sexes.

Adenosine A1 receptors are inhibitory and widespread through the brain while adenosine A2A receptors are excitatory and have the highest density in the basal ganglia, amygdala, and hypothalamus. Adenosine receptor expression levels within the brain are associated with the anxiety response. Low anxiety rats were found to have high A1R and low A2AR expression in the ventral hippocampus, which favors the inhibitory effect of A1R. High anxiety rats expressed low levels of A1R, favoring the excitatory effect of A2AR, increasing glutamate release and causing activation of the HPA axis (Goulart et al. 2025). Our research supports that an imbalance between the inhibitory A1R and the excitatory A2AR expression may impact anxiety‐like behavior. Previous research supports that the adenosine A2A receptor is closely linked with anxiety‐related behavior in humans and animal models (Childs et al. 2008; Yacoubi, Ledent, Ménard, et al. 2000; Jain et al. 1995; Maximino et al. 2011; Rosa et al. 2018; Hughes and Hancock 2016; Hohoff et al. 2010; Fulton et al. 2018; Alsene et al. 2003; Rogers et al. 2010; Goulart et al. 2025). Our research in zebrafish does not show the same pattern between A1AR and A2AR, but suggests that the A2Aa and A2Ab receptors have counteracting effects with A2Ab being more highly expressed in the high stress reactive strain and A2Aa being more highly expressed in the low stress proactive strain. We also found that A2Ab expression, but not A2Aa expression, is positively correlated with anxiety behavior across all tested zebrafish.

Behavioral effects of adenosine receptor expression levels have not been fully investigated. Overexpression of A2A receptors in rats causes depressive‐like behavior and hyperlocomotion (Coelho et al. 2014). Gene expression of A1b, A2Aa, A2Ab, and A2B increased in the zebrafish brain after an acute restraint stressor (Piato, Rosemberg, et al. 2011). This study suggests that A2Aa and A2Ab expression increased due to stress, and A1b expression increased as a compensatory mechanism to maintain equilibrium of excitatory signals between the A2A and A1 receptors. The contrasting A2Aa and A2Ab receptor expression in the zebrafish brain has not been fully investigated but has been noted in previous studies. Following exposure to methionine, mRNA levels of A1 and A2Aa increased while A2Ab levels decreased (Vuaden et al. 2016). Expression of A2Aa increased after exposure to valproic acid while A2Ab expression did not change (Zimmermann et al. 2017). The results from these studies suggest that A2Aa and A2Ab receptor expression may be controlled through separate mechanisms and respond to stressors in distinct ways.

In this study, caffeine treatment decreased expression of *adora1b*, *adora2aa*, and *adora2ab*, independent of strain. This is consistent with the known pharmacodynamics of caffeine as an adenosine receptor antagonist, which can lead to downregulation of receptor mRNA expression through feedback mechanisms (Svenningsson et al. 1999; Ribeiro and Sebastião 2010; Toth and Shenk 1994). The suppression of these genes supports the interpretation that caffeine engaged its molecular targets, leading to behavioral differences. Previous studies have shown that double heterozygous A1R and A2AR mice portray similar effects of chronic caffeine use (5–7 days) (Yang et al. 2009), suggesting that the downregulation of A1 and A2A receptors may be partially responsible for the effects of caffeine. It is also of note that the adenosine receptor downregulation in the current study occurred relatively quickly, within 1 h of acute caffeine treatment. Since examining whole‐brain expression levels can negate spatial transcription patterns, future studies should investigate gene expression of adenosine signaling in specific brain regions.

Adenosine levels are closely regulated through complex processes, including metabolism and transport. Expression and activity of enzymes within the adenosine signaling pathway can impact adenosine concentrations and alter behavioral states. *ada*, which degrades adenosine into inosine, has been linked to anxiety. *ada* knockout mice have increased anxiety behavior (Sauer et al. 2017). Unpredictable chronic stress has been found to decrease *ada* activity and increase extracellular adenosine levels in zebrafish brains, potentially acting as a calming mechanism (Zimmermann et al. 2016). Our study found that *ada* expression is positively correlated with anxiety behavior, although the effect size is moderate (Figure 4). A potential mechanism linking strain differences in adenosine signaling and behavior may be that the reactive strain has reduced adenosine levels. This may be due to elevated expression of *ada*, which degrades adenosine, and slightly reduced expression of *nt5e*, which produces it. The high anxiety behavior observed within the reactive zebrafish strain may partially be caused by low adenosine levels. In addition, caffeine treatment likely acts as a stressor, indicated by increased whole‐body cortisol in zebrafish following exposure (Rosa et al. 2018; Li et al. 2024; K. Wong, Stewart, et al. 2010). We have shown that the caffeine‐treated reactive zebrafish had significantly lower *ada* expression than controls, potentially indicating an increase in adenosine that may act as a calming mechanism in response to caffeine exposure.

We found significant main effects of strain, treatment, and sex on a composite measure of stress behavior. Consistent with prior studies, the reactive strain and females had increased stress behaviors (Wong et al. 2012, 2013, 2015, 2019; Goodman and Wong 2020). Surprisingly, we observed that caffeine treatment resulted in a reduction of stress behaviors. While many prior studies have documented anxiogenic effects of caffeine, this relationship is complex (Jain et al. 1995; Maximino et al. 2011; Rosa et al. 2018; Hughes and Hancock 2016; Li et al. 2024; Egan et al. 2009; Yacoubi, Ledent, Parmentier, et al. 2000). Strain‐ and caffeine concentration‐specific behavioral effects in response to acute caffeine exposure have been noted in prior studies (Rosa et al. 2018; Hughes and Hancock 2016). When investigating the behavioral response to caffeine in wild type and leopard zebrafish populations, the leopard zebrafish had higher baseline cortisol levels and exhibited heighted anxiety responses to caffeine compared to wild type zebrafish (Rosa et al. 2018). In addition, low dose caffeine typically induces stimulatory effects, indicated by increased swimming distance and speed (Yacoubi, Ledent, Ménard, et al. 2000; Egan et al. 2009; Tran et al. 2017; Svenningsson et al. 1997). On the other hand, high doses of caffeine commonly induce anxiety behavior, indicated by decreased swimming distance and increased time frozen (Yacoubi, Ledent, Ménard, et al. 2000; Egan et al. 2009; Tran et al. 2017). While we found that acute caffeine treatment reduced stress behavior, we cannot rule out that chronic exposure would elicit a different behavioral response between the stress coping styles and sexes.

Intriguingly, there was a significant strain by treatment by sex interaction effect on the composite measure of stress behavior. The anxiolytic effect of caffeine treatment was only seen in males with the reactive stress coping style. This strain‐ and sex‐specific effect of caffeine was also seen when examining discrete behaviors where female reactive fish became more anxious after caffeine treatment, while reactive male fish became less anxious but more hyperactive. Our results emphasize the importance of accounting for stress coping style and sex when evaluating the impact of adenosine signaling modulators. Further, it suggests that biases in behavioral stress response characteristic of each stress coping style may be primarily driven by males with the reactive stress coping style. Other studies have found strain‐specific effects of a drug (including ethanol, caffeine, cannabidiol) (Rosa et al. 2018; Hughes and Hancock 2016; Goodman and Wong 2020; Silote et al. 2021). In addition, sex‐specific behavior in response to caffeine has been seen previously in zebrafish (Li et al. 2024), in which acute 300 mg/L caffeine exposure caused female fish to have more freezing behavior and higher whole‐body cortisol compared to male fish, while male fish had increased erratic movement and higher testosterone compared to female fish. In addition, Gene Ontology pathway analysis shows that caffeine may impact steroid hormone biosynthesis, indicating that caffeine may differentially affect stress behavior between the sexes due to altered cortisol and testosterone production (Li et al. 2024). On the other hand, some studies did not find differential effects of sex on anxiety behavior following caffeine treatment (Hughes and Hancock 2016; Wilson et al. 2024). To our knowledge, this is the first study showing both strain‐ and sex‐dependent effects of caffeine.

## 5 Conclusion

Overall, our findings reinforce the central role of coping style in shaping both behavioral and molecular responses to stress and neuromodulatory compounds. Acute caffeine exposure exerts coping style‐ and sex‐dependent effects on anxiety‐like behavior in zebrafish, with reactive individuals, particularly males, showing the greatest sensitivity. The accompanying changes in adenosine receptor and *ada* expression demonstrate a possible molecular mechanism underlying these behavioral differences, highlighting the adenosine signaling pathway as a promising target for understanding individual variability in anxiety. We hypothesize that a combined effect of adenosine receptor expression, extracellular adenosine levels, and downstream adenosine signaling contributes to how the adenosine signaling pathway impacts anxiety‐related behavior. Further, this mechanism can explain, in part, the different biases in behavioral and physiological responses to stress between the stress coping styles. Future studies incorporating region‐specific brain analyses, chronic caffeine exposure paradigms, or more targeted pharmacological manipulations will help clarify the causal links between adenosine signaling, stress coping style, and anxiety behavior.

## Author Contributions

**Sydney E. Klucas**: conceptualization, funding acquisition, writing – original draft, methodology, formal analysis, writing – review and editing, investigation, visualization. **Ryan Y. Wong**: conceptualization, funding acquisition, writing – review and editing, formal analysis, supervision, resources.

## Ethics Statement

This study was approved by the Institutional Animal Care and Use Committee (IACUC) of the University of Nebraska at Omaha (17‐070‐09‐FC).

## Conflicts of Interest

The authors declare no conflicts of interest.

## Supporting information




**Supplementary Material**: brb371445‐sup‐0001‐SuppMat.pdf

## Data Availability

All data generated or analyzed during this study are included in Supporting Information files.
