# Supplementary material for: Differential Adenosine Signaling and Effects of Acute Caffeine Exposure on Alternative Stress Coping Styles in Zebrafish (Danio rerio)
Source: Brain Behav. 2026 Apr 29;16(5):e71445. doi: 10.1002/brb3.71445 (PMC13128979; doi:10.1002/brb3.71445)
Supplement: Supplementary file 1 — Supplementary Material: brb371445‐sup‐0001‐SuppMat.pdf [file BRB3-16-e71445-s001.pdf]

Differential adenosine signaling and effects of acute caffeine exposure on alternative stress coping styles in zebrafish (*Danio rerio*)

Sydney E. Klucas and Ryan Y. Wong*

Author Affiliation: Department of Biology, University of Nebraska at Omaha

* Correspondence: rwong@unomaha.edu

Supplemental Methods, Results, and Data

To identify a biologically relevant dose and behavioral trial length applicable to both proactive and reactive zebrafish, we acutely treated with 0 (n = 16 of each coping style), 50 (n = 8 of each coping style), 100 (n = 8 of each coping style), or 150 mg/L caffeine concentrations (n = 8 of each coping style) for 15 minutes and then quantified stress behaviors (distance swam, time spent in bottom half of tank, swimming velocity, time frozen) in the novel tank diving test for 6 minutes. We used a generalized linear model (GLZM) in SPSS (Version 29) to examine main effects of strain (reactive, proactive), caffeine concentration (0, 50, 100, 150 mg/L caffeine) and strain by treatment interaction effect on distance swam, time spent in bottom half of tank, swimming velocity. For time frozen we used GLZM to examine the main effects of strain (reactive, proactive), caffeine concentration (0, 50 mg/L caffeine) and strain by treatment interaction effect. To evaluate the direction of effects, we examined the simple main effects within each GLZM. Overall, caffeine concentration had minimal effects on stress behavior of proactive zebrafish. However, in the reactive strain, exposure to 50 mg/L caffeine increased stress behavior and 100 mg/L caffeine increased locomotion relative to controls.

There was no main effect of caffeine concentration on distance swam (Wald $\chi^2 = 5.765$, $p = .124$), time spent in the bottom half (Wald $\chi^2 = 3.863$, $p = .277$), swimming velocity (Wald $\chi^2 = 5.682$, $p = .128$), and time spent frozen (Wald $\chi^2 = 2.122$, $p = .145$) (Supplementary Figure 1). There were significant main effects of strain on distance swam (Wald $\chi^2 = 114.515$, $p < .001$), time spent in the bottom half (Wald $\chi^2 = 30.857$, $p = 2.7 * 10^{-8}$), swimming velocity (Wald $\chi^2 = 114.453$, $p < .001$), and time spent frozen (Wald $\chi^2 = 118.24$, $p < .001$). There were significant strain by treatment interaction effects on distance swam (Wald $\chi^2 = 9.045$, $p = .029$), swimming velocity (Wald $\chi^2 = 8.706$, $p = .033$), and time spent frozen (Wald $\chi^2 = 5.038$, $p = .025$). Reactive fish treated with 50 mg/L caffeine spent significantly more time frozen than reactive controls ($p = .009$). There was a trend for reactive fish treated with 50 mg/L caffeine to swim less distance ($p = .122$) and with slower velocity ($p = .138$) than reactive controls. There were no significant differences between 50 mg/L caffeine treated and control proactive fish for distance swam ($p = .277$) and swim velocity ($p = .282$). Reactive fish treated with 100 mg/L caffeine swam significantly higher distance ($p = .014$) and with faster swimming velocity ($p = .015$) than reactive controls. Treatment of 150 mg/L caffeine did not significantly impact the behavior of reactive fish. Neither 100 mg/L nor 150 mg/L caffeine treatment significantly impacted the behavior of proactive fish. There was not a significant strain by treatment interaction effect on time spent in the bottom half (Wald $\chi^2 = 5.525$, $p = .137$).

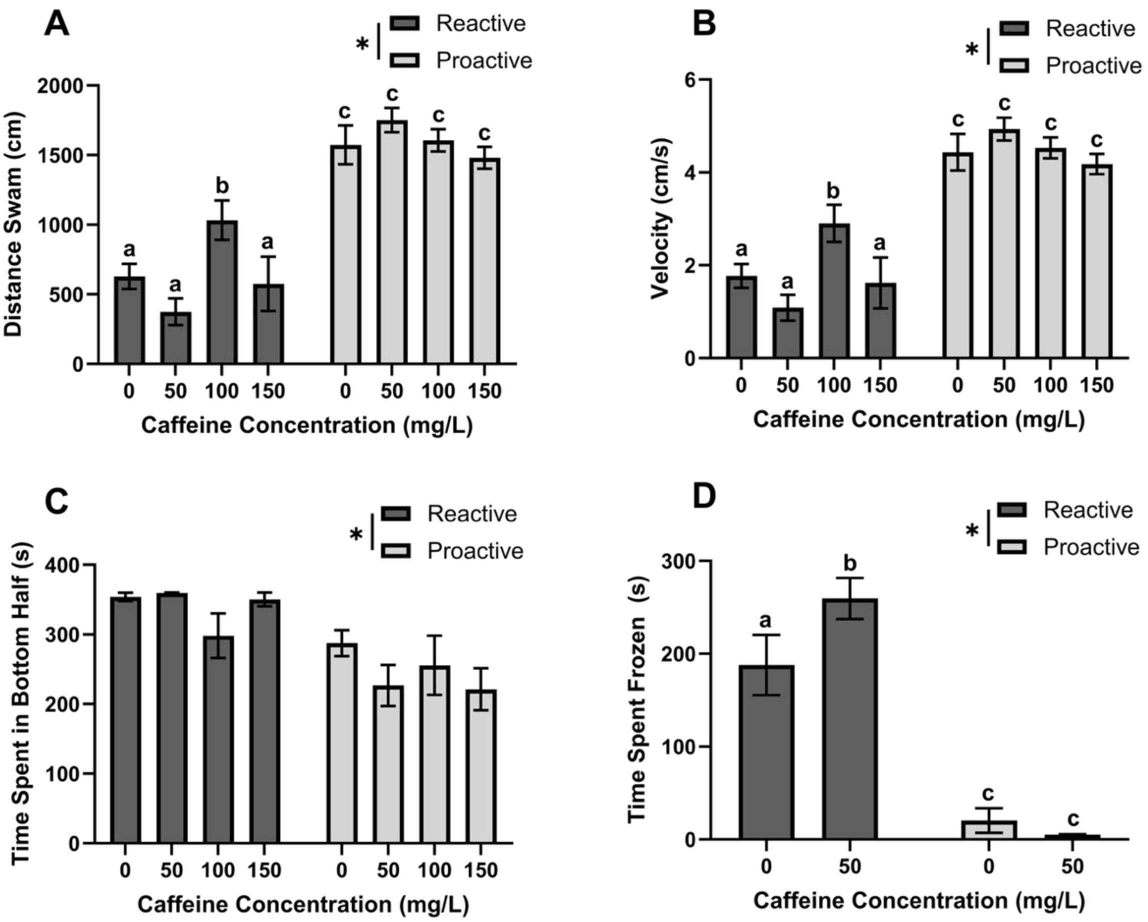

Supplemental Figure 1. Dose response analysis at each caffeine concentration (0, 50, 100, 150 mg/L) for behavioral variables: distance swam (A), average velocity (B), time spent in bottom half (C), and time spent frozen (D). Data shown are mean ± 1 SEM. Significant strain differences are indicated by an asterisk ($p \leq 0.05$), and differences between groups are indicated by different lower-case letters ($p \leq 0.05$).

Gene	Primer Conc.	Amplicon Length (bp)	Forward Primer	Reverse Primer
adora1b	5 pmol	95	5’-GTCCAGTCATTCGGAACCCA-3’	5’-GCCAGCTAATTGCGAACAGG-3’
adora2aa	1 pmol	121	5’-GTTGCCTGTTCATCGCCTG-3’	5’-ACTAGGCTGTTGTACCTAAGCG-3’
adora2ab	2.5 pmol	89	5’-CTTTTCGCAGTTTGCTGGCT-3’	5’-ATGACCCAATCCTGAGGTCG-3’
adora2b	5 pmol	76	5’-AGTCTTCTAGCGGTTGCCAT-3’	5’-TACCTGTGACCAGCTCCCTA-3’
ada	5 pmol	134	5’-CGGCGAGGAATCAGTCTACC-3’	5’-TCTCTGTCCCCTGCAATGAC-3’
nt5e	5 pmol	117	5’-CTGGTCCCCAACAAGGTGTA-3’	5’-TATATCCAGATCACCGCTGTCG-3’
ef1a	5 pmol	150	5’-CCTCTTGGTCGCTTTGC-3’	5’-GGTGTGATTGAGGGAAATTCA-3’

Supplemental Table 1. qRT-PCR Primers

Strain	Caffeine Conc. (mg/L)	Distance Swam (cm)	Velocity (cm/s)	Time Frozen (s)	Bottom Time (s)
Reactive	0	793.503	2.24331	Not Collected	359.4252
Reactive	0	543.777	1.53139	Not Collected	360.0587
Reactive	0	210.613	0.592462	Not Collected	360.0593
Reactive	0	797.597	2.24789	Not Collected	360.0592
Reactive	0	1436.08	4.04277	Not Collected	311.8779
Reactive	0	236.778	0.667065	Not Collected	360.0581
Reactive	0	131.379	0.369921	Not Collected	360.059
Reactive	0	830.862	2.35426	Not Collected	359.3916
Reactive	0	1147.07	3.22858	13.5468	Not Collected
Reactive	0	676.277	1.91353	96.3294	Not Collected
Reactive	0	671.123	1.88896	224.524	Not Collected
Reactive	0	257.909	0.725643	278.878	Not Collected
Reactive	0	648.212	1.82447	270.069	Not Collected
Reactive	0	261.637	0.736963	194.727	Not Collected
Reactive	0	541.602	1.52641	249.315	Not Collected
Reactive	0	879.767	2.47994	174.674	Not Collected
Reactive	50	128.703	0.473979	193.46	360.059
Reactive	50	263.703	0.74111	290.79	360.05849
Reactive	50	418.292	1.17667	272.538	360.05853
Reactive	50	728.169	2.04991	218.585	360.0587
Reactive	50	542.037	1.52649	255.121	360.0589
Reactive	50	79.5004	0.223974	351.517	360.059
Reactive	50	101.077	0.28428	322.655	360.059
Reactive	50	737.93	2.21868	171.738	356.32148
Reactive	100	991.905	2.78869	Not Collected	360.0597
Reactive	100	336.307	0.94498	Not Collected	360.05903
Reactive	100	1613.47	4.53791	Not Collected	231.4972
Reactive	100	1120.18	3.14992	Not Collected	329.7619
Reactive	100	1405.14	3.95903	Not Collected	108.2413
Reactive	100	1135.93	3.19181	Not Collected	275.0076
Reactive	100	1000.16	2.81085	Not Collected	360.0582
Reactive	100	654.227	1.84106	Not Collected	360.0583
Reactive	150	1174.39	3.3061	Not Collected	359.959
Reactive	150	521.901	1.47172	Not Collected	360.05924
Reactive	150	1498.08	4.2189	Not Collected	281.7804
Reactive	150	0	0	Not Collected	360.059
Reactive	150	114.97	0.324571	Not Collected	360.05906
Reactive	150	881.492	2.48386	Not Collected	360.05956
Reactive	150	148.455	0.418631	Not Collected	360.059
Reactive	150	264.697	0.743764	Not Collected	360.0592
Proactive	0	592.747	1.6693	Not Collected	355.1877
Proactive	0	2066.76	5.81823	Not Collected	247.6469
Proactive	0	1092.26	3.07373	Not Collected	336.3688
Proactive	0	1379.16	3.90602	Not Collected	283.5491
Proactive	0	1394.47	3.96589	Not Collected	309.5754
Proactive	0	1229.28	3.46712	Not Collected	271.4038
Proactive	0	2149.78	6.06279	Not Collected	307.5397
Proactive	0	1670.31	4.71725	Not Collected	188.9217
Proactive	0	998.438	2.82242	17.6509	Not Collected
Proactive	0	1627.18	4.57218	6.30628	Not Collected
Proactive	0	1786.55	5.02516	3.70369	Not Collected
Proactive	0	2003.32	5.63647	7.00697	Not Collected
Proactive	0	903.446	2.54381	112.245	Not Collected
Proactive	0	2771.52	7.79491	3.77043	Not Collected
Proactive	0	2058.11	5.79934	3.97062	Not Collected
Proactive	0	1442.78	4.06928	8.37502	Not Collected
Proactive	50	1837.18	5.18459	5.47212	228.4277
Proactive	50	1840.82	5.17926	3.7037	93.9604
Proactive	50	1794.72	5.03822	6.70668	356.5555
Proactive	50	1665.05	4.69178	3.87052	301.3007
Proactive	50	2057.4	5.79188	3.90389	151.084
Proactive	50	1284.16	3.63045	6.97361	215.3814
Proactive	50	1545.51	4.34757	6.23956	269.2681
Proactive	50	1986.62	5.59263	3.93727	197.8639
Proactive	100	1323.68	3.72987	Not Collected	346.98

Proactive	100	1828.27	5.14782	Not Collected	16.31626
Proactive	100	1892.85	5.34774	Not Collected	128.9619
Proactive	100	1677.95	4.73256	Not Collected	335.401
Proactive	100	1706.51	4.79867	Not Collected	311.177
Proactive	100	1441.55	4.07309	Not Collected	263.8629
Proactive	100	1680.68	4.7376	Not Collected	296.1285
Proactive	100	1293.45	3.66328	Not Collected	346.2787
Proactive	150	1467.67	4.13132	Not Collected	321.3208
Proactive	150	1508.84	4.26604	Not Collected	358.5907
Proactive	150	1251.17	3.57256	Not Collected	98.83188
Proactive	150	1343.92	3.78475	Not Collected	191.8914
Proactive	150	1299.11	3.66407	Not Collected	235.33425
Proactive	150	1675.88	4.72719	Not Collected	178.3778
Proactive	150	1917.84	5.40103	Not Collected	155.1547
Proactive	150	1383.28	3.89122	Not Collected	230.4635

Supplemental Table 2. Dose Response Behavior Data.

ID	Strain	Treatment	Sex	PC Score	Distance Swam (cm)	Velocity (cm/s)	Time Frozen (s)	Top Transitions	Bottom Time (s)	*adora1b / ef1a*	*adora2aa / ef1a*	*adora2ab / ef1a*	*adora2b / ef1a*	*ada / ef1a*	*nt5e / ef1a*
R1C	Reactive	Control	F	-1.525	163.89	0.46	240.94	0	360.00	2.23	2.20	2.60	2.60	2.35	1.34
R2C	Reactive	Control	F	-1.440	34.99	0.49	336.71	0	73.91	2.10	2.31	2.19	1.99	1.76	0.54
R3C	Reactive	Control	M	-1.852	70.30	0.20	353.39	0	360.00	4.60	1.35	1.70	2.00	1.86	1.60
R4C	Reactive	Control	M	-1.094	446.81	1.26	151.99	0	360.00	1.58	1.10	1.84	1.32	2.25	0.39
R5C	Reactive	Control	M	0.543	1532.19	4.31	10.21	28	294.74	3.10	1.89	1.54	2.61	1.05	0.02
R6C	Reactive	Control	F	-1.680	165.48	0.47	311.42	0	360.00	2.62	1.36	1.63	1.94	0.96	0.62
R7C	Reactive	Control	M	-1.647	134.56	0.38	284.73	0	360.00	1.89	1.57	1.61	2.52	1.67	1.26
R8C	Reactive	Control	M	0.046	1218.61	3.44	12.08	12	325.60	2.55	1.63	1.81	2.54	1.67	0.59
R9C	Reactive	Control	F	-0.143	1225.91	3.45	13.95	0	360.00	2.73	2.17	2.04	3.02	1.15	0.81
R10C	Reactive	Control	F	-1.227	403.85	1.14	196.06	0	360.00	1.70	2.12	2.50	2.23	2.10	0.96
R11C	Reactive	Control	F	-1.099	465.81	1.31	187.12	3	343.24	1.83	1.71	1.71	2.34	1.75	1.07
R12C	Reactive	Control	M	0.546	1589.02	4.48	4.64	21	270.74	1.65	1.85	1.42	2.17	1.18	0.66
R13C	Reactive	Control	M	-0.629	797.24	2.25	137.54	6	303.67	1.76	1.95	1.62	2.73	1.30	1.09
R14C	Reactive	Control	M	-1.155	340.57	0.96	203.14	2	264.77	1.23	2.75	1.45	2.52	1.92	0.05
R15C	Reactive	Control	M	0.039	1258.65	3.61	34.70	8	283.45	2.80	1.81	1.49	2.13	1.45	1.35
P1C	Proactive	Control	M	-0.023	1020.30	2.87	15.75	18	300.91	1.93	2.08	1.60	2.26	1.84	1.53
P2C	Proactive	Control	M	0.522	1509.32	4.25	5.24	24	264.87	1.56	2.74	1.56	1.95	1.57	1.37
P3C	Proactive	Control	M	0.446	1254.11	3.54	10.28	27	176.02	1.90	2.93	3.00	2.92	1.63	0.90
P4C	Proactive	Control	F	1.189	1846.87	5.21	5.51	55	261.57	1.33	1.88	1.39	1.94	0.80	1.34
P5C	Proactive	Control	M	0.182	1349.12	3.79	57.76	22	319.49	2.13	2.06	1.79	2.24	1.12	1.65
P6C	Proactive	Control	F	0.151	1386.55	3.90	46.55	17	340.01	1.49	1.93	1.39	2.04	1.09	1.43
P7C	Proactive	Control	F	0.948	1439.60	4.05	4.24	57	204.78	1.87	2.35	2.65	2.34	0.99	1.65
P8C	Proactive	Control	M	0.711	1778.36	5.02	5.31	25	304.18	1.94	2.37	1.61	1.91	1.28	1.71
P9C	Proactive	Control	M	0.582	1434.53	4.04	10.51	35	267.14	1.72	2.44	1.35	2.29	1.11	1.60
P10C	Proactive	Control	F	-0.272	1047.33	2.94	61.16	8	339.08	1.40	1.85	1.68	2.32	1.39	0.66
P11C	Proactive	Control	M	0.701	1704.02	4.80	5.34	30	312.39	1.59	2.24	1.44	2.17	1.22	1.00
P12C	Proactive	Control	M	1.060	1771.17	4.99	4.80	42	185.03	0.93	2.12	1.56	1.76	0.92	0.67
P13C	Proactive	Control	F	0.379	1406.32	3.95	20.25	23	274.52	0.71	1.70	0.99	1.23	0.65	0.97
P14C	Proactive	Control	F	-0.233	766.30	2.16	38.20	18	259.40	1.23	2.67	1.65	2.03	1.45	0.55
P15C	Proactive	Control	M	0.381	1640.14	4.62	6.51	10	326.29	1.65	2.56	1.34	2.04	1.11	1.12
R1T	Reactive	Treated	F	1.213	1901.27	5.36	3.87	36	89.26	1.38	2.47	1.34	1.79	1.05	1.33
R2T	Reactive	Treated	F	-1.810	87.90	0.25	340.97	0	360.00	1.57	1.60	1.10	2.79	1.33	0.73
R3T	Reactive	Treated	F	-1.601	143.32	0.40	267.61	0	360.00	1.79	1.63	1.65	2.02	0.96	1.06
R4T	Reactive	Treated	F	-1.823	77.45	0.22	342.98	0	360.00	1.38	1.01	1.72	2.18	0.94	0.02
R5T	Reactive	Treated	M	0.743	1654.62	4.66	43.38	31	183.72	1.96	1.56	2.44	2.88	0.91	1.13
R6T	Reactive	Treated	M	0.686	2143.72	6.06	71.37	12	321.43	1.99	1.79	2.42	2.20	1.19	0.67
R7T	Reactive	Treated	F	-1.847	77.13	0.22	353.79	0	360.00	2.21	2.07	1.63	2.75	1.11	0.93
R8T	Reactive	Treated	M	0.831	2204.71	6.20	1.87	10	347.75	1.89	1.96	1.86	1.59	1.33	1.41
R9T	Reactive	Treated	M	-0.398	839.71	2.37	134.80	11	197.66	2.31	1.92	2.33	1.98	2.90	1.39
R10T	Reactive	Treated	M	0.677	2144.21	6.07	3.47	2	353.59	1.39	1.89	1.09	1.72	1.46	1.28
R11T	Reactive	Treated	M	0.361	1214.46	3.42	8.54	15	98.97	1.30	1.51	1.84	1.92	1.59	1.23
R12T	Reactive	Treated	M	0.573	1722.53	4.85	6.71	21	335.07	1.62	1.52	1.43	2.12	1.13	0.53
R13T	Reactive	Treated	F	-1.489	313.49	0.88	320.56	7	357.83	1.49	1.74	1.65	2.58	1.00	1.00
R14T	Reactive	Treated	F	-0.260	742.26	2.09	129.60	13	47.28	1.88	2.00	1.66	2.49	0.97	1.47
R15T	Reactive	Treated	M	-0.469	589.71	1.67	142.58	4	0.83	1.36	1.88	1.69	1.83	1.82	0.81
P1T	Proactive	Treated	M	0.381	1306.00	3.68	8.88	25	246.32	1.44	1.82	1.17	2.13	0.92	0.85
P2T	Proactive	Treated	M	0.062	1392.04	3.93	14.21	5	355.33	1.44	1.87	1.99	1.80	1.32	2.14
P3T	Proactive	Treated	F	1.001	1760.51	5.05	5.64	40	215.05	1.10	2.40	1.16	2.50	1.50	1.94
P4T	Proactive	Treated	M	0.942	1794.09	5.07	5.27	30	176.11	1.41	1.76	1.00	1.77	1.13	0.80
P5T	Proactive	Treated	M	-0.058	845.57	2.38	19.29	23	254.80	2.09	1.88	1.88	2.38	1.68	1.01
P6T	Proactive	Treated	M	1.548	2070.69	5.83	4.30	63	202.24	1.56	1.85	1.61	1.99	0.88	0.73
P7T	Proactive	Treated	M	1.072	1950.08	5.49	3.50	35	226.83	1.65	1.85	1.94	2.59	1.04	0.77
P8T	Proactive	Treated	F	0.616	1532.23	4.31	5.47	33	294.27	1.28	1.81	1.47	1.87	0.88	0.61
P9T	Proactive	Treated	F	1.118	2146.56	6.04	4.54	33	301.24	1.63	1.71	1.09	2.11	0.71	0.54
P10T	Proactive	Treated	M	0.420	1438.01	4.07	26.73	29	314.56	0.92	1.99	1.16	1.49	0.97	0.79
P11T	Proactive	Treated	F	0.183	1272.06	3.59	10.74	18	312.65	1.24	2.37	1.28	1.92	0.73	0.55
P12T	Proactive	Treated	F	-1.054	664.26	1.88	221.92	1	358.33	1.77	2.06	1.82	1.76	2.83	1.19
P13T	Proactive	Treated	M	0.939	1823.00	5.14	4.77	37	269.14	1.99	2.04	1.08	1.96	1.46	1.29
P14T	Proactive	Treated	M	1.133	1656.22	4.67	8.01	62	245.11	1.85	1.90	1.38	2.07	1.21	0.03
P15T	Proactive	Treated	M	1.904	2206.66	6.23	2.70	85	230.04	2.01	1.61	1.08	1.55	1.21	1.22

Supplemental Table 3. Behavior and Gene Expression Data.
